# Supplementary material for: A Putative NADPH Oxidase Gene in Unicellular Pathogenic Candida glabrata Is Required for Fungal ROS Production and Oxidative Stress Response
Source: J Fungi (Basel). 2023 Dec 27;10(1):16. doi: 10.3390/jof10010016 (PMC10817436; doi:10.3390/jof10010016)
Supplement: Supplementary file 1 [file jof-10-00016-s001.zip › jof-2750953-supplementary.pdf]

Supplementary Materials:

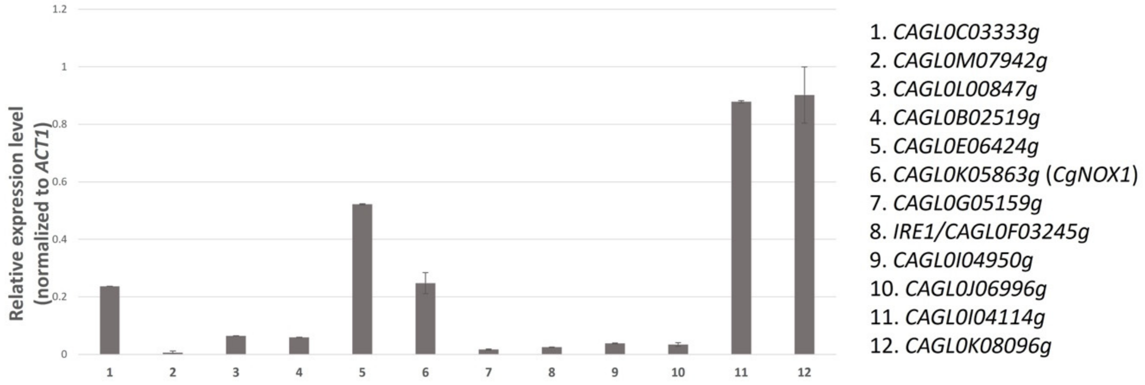

Figure S1. Real-time quantitative RT-PCR analysis of mRNA expression of twelve *NOX* candidate genes in wild-type *C. glabrata*. The *ACT1* transcript level in *C. glabrata* was used for normalization, with the *ACT1* expression level set as 1. Reported values indicate the mean ± SD of at least three independent experiments.

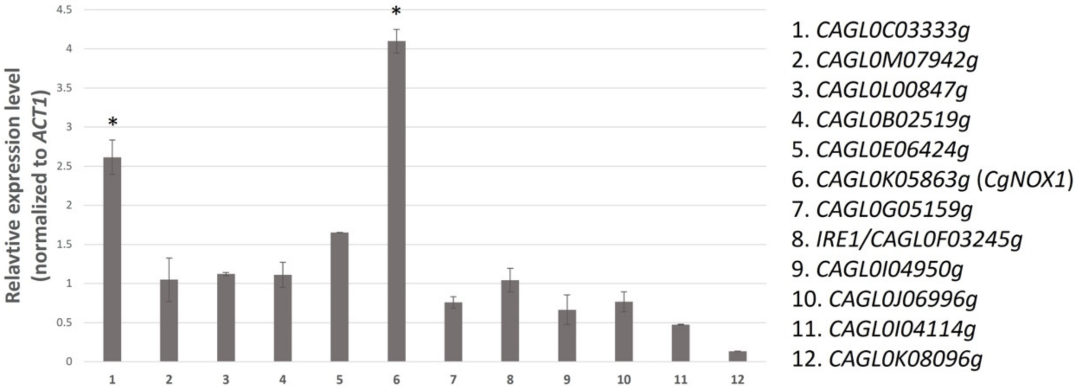

Figure S2. Real-time quantitative RT-PCR analysis of mRNA expression of twelve *NOX* candidate genes in wild-type *C. glabrata* during co-incubation with hepatic cells. *ACT1* transcript level in *C. glabrata* was used for normalization. The relative expression of each gene was compared with that of the respective single-cultured group. Reported values indicate the mean ± SD of at least three independent experiments. *$p<0.05$ compared with the respective single-cultured group.

Table S1. Primers for real-time PCR.

Primer name	Sequence 5'-3'
ACT1_F	CCTACGAATTGCCAGATGGT
ACT1_R	ACAGATGGGTGGAACAAAGC
3333_F	AGGACCAAGAACTTGTTCGC
3333_R	TCCCAATTGCCAACAATGCC
7942_F	ACTTCCGCCACCATTTTGTG
7942_R	AAATTGCCCTGCGAACCATG
0847_F	AGATTTTGGCGACGTTCTCG
0847_R	TTCAACAGTGCCTTCACAGC
2519_F	ACTGCGCTTTCTGGTGAATG
2519_R	TCAATGTCTGGTCTTCGAGCTC
6424_F	TACGGCAGTAAGACACCATCTG
6424_R	GGTGTCGTCCTTGTCAACAAAG
5863_F	TGCGAACCGAATTGCCATAG
5863_R	ACTGGCTGCTGTTTTTGGTC
5159_F	CTTCTACCGTTAGCCTTGCATG
5159_R	TGCGTGATATCCTCCCTTCTTG
IRE1-F	AATAGACCCAGCGCTGAAGTAG
IRE1-R	TCGCGGGTTTCAATTTCCAG
4950-F	TGCCGGACAGTTATGGGTAAAG
4950-R	TCGCTCGCAACTTTTGAACC
6996-F	TCGTAGTTCCAGCCAAGAGATG
6996-R	TTGTTCGGACGGCAAATCTG
4114-F	AGAAGGCATTGCTTGAGCTG
4114-R	TGGATCAGTTGCAGTGGAGTC
8096-F	AAAAGAGGCGCAGGAGATAGAC
8096-R	AGCATCAACCCAAGCACAAG

Table S2. Protein identity of NOX candidates using BLAST analysis.

NOX candidates in *Candida glabrata*	Protein identity to *Sc*Yno1p/*Sc*Aim14p
CAGL0C03333g	22.22%
CAGL0M07942g	24.08%
CAGL0L00847g	29.63%
CAGL0B02519g	37.14%
CAGL0E06424g	34.62%
CAGL0K05863g (CgNOX1)	40.94%
CAGL0G05159g	/
IRE1/CAGL0F03245g	/
CAGL0I04950g	/
CAGL0J06996g	/
CAGL0I04114g	29.17%
CAGL0K08096g	/
